# Clinical and Genetic Analysis of a Patient With Coexisting 17a-Hydroxylase/17,20-Lyase Deficiency and Moyamoya Disease

**DOI:** 10.3389/fgene.2022.845016

**Published:** 2022-08-30

**Authors:** Jiaming Huang, Danli Zhou, Nan Dong, Chenzhao Ding, Yan Liu, Fangping Li

**Affiliations:** ^1^ Department of Endocrinology, The Seventh Affiliated Hospital, Sun Yat-sen University, Shenzhen, China; ^2^ Department of Scientific Research Center, The Seventh Affiliated Hospital, Sun Yat-sen University, Shenzhen, China

**Keywords:** 17a-hydroxylase/17, 20-lyase deficiency, moyamoya disease, CYP17A1 gene, PCNT gene, CNOT3 gene, HLA-DRB1 gene

## Abstract

17a-Hydroxylase/17,20-lyase deficiency (17OHD) is caused by pathogenic mutations in *CYP17A1*. Female patients present with hypertension, hypokalemia, and sexual infantilism while males present with sex development disorder. Moyamoya disease (MMD) is a chronic cerebrovascular disease that frequently results in intracranial ischemia or hemorrhage. The present study describes a case of 17OHD and MMD in a 27-year-old phenotypically female (46, XY) patient and discusses the clinical features and characteristics of her genetic defect. Clinical, hormonal, radiological, and genetic analyses were performed and blood samples were collected for whole-exome sequencing (WES). The results of the WES revealed a homozygous intronic mutation (c.297+2T>C) in *CYP17A1*, which led to combined 17a-hydroxylase/17,20-lyase deficiency, as well as novel variants in *PCNT* and *CNOT3* that might lead to MMD. To our knowledge, this study is the first to describe 17OHD accompanied by MMD. While several cases have previously described patients with 17OHD with histories of cerebral hemorrhage or cerebral ischemia, a correlation in genetic levels between 17OHD and MMD was not found. The risk of cerebrovascular accidents should be considered in patients with 17OHD and hypertension. Cerebrovascular examination in patients with 17OHD may be beneficial for the prevention of life-threatening intracranial vascular disease.

## 1 Introduction

17a-Hydroxylase/17,20-lyase (17OHD) deficiency (OMIM 202110) is a rare form of congenital adrenal hyperplasia (CAH). It is an autosomal recessive disease caused by a gene defect in cytochrome P450 family 17 subfamily A member 1 (*CYP17A1*). *CYP17A1* is located on chromosome 10q24.3 (Sparkes et al., 1991). The full length of the gene is about 6.6 KB and includes eight exons and seven introns (Picado-Leonard and Miller, 1987). This gene transcribes an identical mRNA encoding cytochrome P450c17 enzyme in both adrenal glands and gonads (Chung et al., 1987). Mutations in *CYP17A1* lead to functional deficiency of the P450c17 enzyme, which prevents the synthesis of glucocorticoids and sex hormones and leads to halocorticoid accumulation. The main clinical manifestations are hypertension, hypokalemia, female sexual infantile and primary amenorrhea, and male sex development disorder. The true incidence of 17OHD is unknown but may be approximately 1 in 50,000 (El-Maouche et al., 2017). Most known mutations appear to be novel, with 148 new mutations characterized in the Human Gene Mutation Database.

Moyamoya disease (MMD) is a chronic and progressive occlusive cerebrovascular disease of unknown etiology. MMD mainly manifests as stenosis or occlusion of the distal ends of bilateral internal carotid arteries and their large branches, accompanied by an abnormal neovascularization network (moyamoya vessels) at the base of the skull (“moyamoya” is the Japanese term for a “puff of smoke”), as described by Suzuki and Takaku (1969). The prevalence of MMD in Japan is 10.5/100,000, while the incidence is 0.94/100,000 (Baba et al., 2008). The incidence rate in Nanjing from 2000 to 2007 was 3.92/100,000 (Miao et al., 2010). The clinical manifestations of MMD mainly fall into two categories: 1) hemorrhage and 2) ischemia. The onset ages of MMD are approximately 5 and 40 years (Miao et al., 2010). Epidemiological studies show that MMD mainly occurs in Asian countries. Nearly 10% of patients in Asian countries have a family history of MMD, indicating the important role of genetic factors in its pathogenesis (Bersano et al., 2016).

The coexistence of 17OHD and MMD in one person is extremely rare, with only one previous case reported worldwide (Gao et al., 2019). This study reports the case of a 28-year-old patient with clinical features of 17OHD and MMD is reported, and the characteristics of her genetic defect were analyzed.

## 2 Materials and Methods

### 2.1 Patient

The patient was raised as a girl and evaluated for primary amenorrhea, hypokalemia (3.0 mmol/L), and hypertension (160/120 mmHg) at 20 years of age. She was 158 cm tall, and the physical examination showed that she was a prepuberal girl with no breast development and normal infantile female external genitalia, but with a blind-ending vagina. Her karyotype was 46, XY, sex-determining region Y gene (*SRY*): positive. Ultrasound (US) imaging and magnetic resonance imagining (MRI) revealed the absence of a uterus and ovaries and bilateral adrenal hyperplasia. She had an elder sister who died of asphyxiation at birth and has a healthy younger brother. No consanguinity between her parents had occurred and no relevant family history of hypertension was reported. Examination showed decreased serum cortisol, K^+^, and sex steroid levels but increased serum basal adrenocorticotropic hormone (ACTH), luteinizing hormone (LH), follicle-stimulating hormone (FSH), and aldosterone levels (Table 1). According to the clinical manifestations (disorder of sex development with 46, XY karyotype, hypertension, adrenal hyperplasia) and laboratory examination (hypokalemia, disordered synthesis of cortisol and sex hormones, high ACTH and gonadotropin levels), the patient was diagnosed with 17OHD and treated with oral dexamethasone (0.375 mg once daily). Her blood pressure and potassium were normal while undergoing treatment.

**TABLE 1:** Hormonal findings before treatment with dexamethasone.

Hormonal findings	Results	References
ACTH at 8 AM	127↑	5–46 (pg/ml)
LH	28.21↑	1.24–8.62 (IU/L)
FSH	72.44↑	1.27–19.26 (IU/L)
T	1.17↓	6.07–27.10 (nmol/L)
E2	2.00↓	20–75 (ng/L)
DHEA-S	44↓	100–600 (ng/ml)
P	16.22↑	0.31–1.52 (ug/L)
17-OHP	0.45	0.61–3.34 (ng/ml)
Cortisol at 8 AM	35.48↓	138–690 (nmol/L)
Aldo supine	220↑	29.4–161.5 (ng/ml)
PRA supine	0↓	0.15–2.33 (ng/ml/h)
K^+^	3.00↓	3.5–5.2 (mmol/L)

T, testosterone; E2, estradiol; DHEA-S, dehydroepiandrosterone sulfate; P, progesterone; ACTH, adrenocorticotropic hormone; LH, luteinizing hormone; FSH, follicular stimulating hormone; 17-OHP, 17-hydroxyprogesterone; K, Blood potassium; Aldo, aldosterone; PRA, plasma renin activity.

However, the patient stopped treatment after 1 year due to significant weight gain. Her blood pressure ranged from 150/110 mmHg to 160/120 mmHg after withdrawal.

At 25 years of age, the patient suffered a sudden loss of consciousness and blurred vision twice upon awakening. Cranial computerized tomography angiography (CTA) showed an A1 segment aneurysm of the left anterior cerebral artery. Digital subtraction angiography (DSA) and interventional embolization of the left anterior communication artery aneurysm were performed. During the operation, the left anterior communicating aneurysm was confirmed and bilateral middle cerebral artery occlusion was found. The middle cerebral artery supply area was compensated by bilateral anterior and posterior cerebral arteries and moyamoya vessels were seen. According to the guidelines for the diagnosis of MMD (Willis. and Diseases., 2012), the patient was also diagnosed with MMD. Ophthalmic examination showed a left visual field residual temporal inferior visual island, and a relatively dark spot in the center of the right visual field. A pelvic MRI could not locate a uterus, ovaries, or male genitalia. After the operation, the patient was prescribed hydrocortisone (20 mg at 8 am, 10 mg at 4 pm) and nifedipine (Adalat 30 mg once daily) for 3 months, after which she regularly took dexamethasone (0.375 mg once daily) and nifedipine (Adalat 30 mg once daily) and achieved satisfactory control of her hypertension.

At 27 years of age, the patient came to our hospital for reexamination without presenting any discomfort. Physical examination showed a height of 167 cm, weight of 55.5 kg, and blood pressure of 129/95 mmHg. The patient still showed a lack of pubertal development, with immature breasts and no pubic or axillary hair. Her potassium levels were normal. Her bone age was 13 years at a chronological age of 27 years. Ophthalmology showed optic atrophy and exotropia in her left eye and refractive ametropia in her right eye. The patient’s treatment remained dexamethasone (0.375 mg once daily) and nifedipine (Adalat 30 mg once daily). As the patient’s gender selection was female, she should have received estrogen supplement therapy. However, considering the possibility of the development of malignancy of the testes, laparoscopic surgery was suggested to find and remove any testicular tissue. However, the patient refused the surgery.

### 2.2 Genetic Analysis

Written informed consent was obtained. Peripheral blood leukocyte samples from the patient and her father were collected in an ethylenediaminetetraacetic acid (EDTA) anticoagulant tube. Whole-exome sequencing (WES), including high-throughput sequencing and target region capture technology, were used for gene detection. Sanger sequencing was used to verify the patient and her father. Some experimental procedures were performed by the BGI Clinical Laboratory Center.

#### 2.2.1 Target Region Capture and Sequencing

Genomic DNA (gDNA) was extracted from the patient’s peripheral blood leukocytes. The gDNA was fragmented and collected by magnetic beads, and a single individual DNA library was constructed. The DNA was then captured and enriched on a BGI V4 chip on the exon and adjacent splicing region of the target gene and finally sequenced with PE100+100 on an MGISEQ-2000 instrument.

#### 2.2.2 Data Analysis

Duplicate sequences were removed and aligned to the human genome reference (hg19) using the Burrows–Wheeler aligner (BWA) Multi-Vision software package (Li and Durbin, 2009). GATK software (McKenna et al., 2010) was used to detect single-nucleotide variants (SNVs) and indels. All SNVs and indels were filtered and estimated via multiple databases, including HapMap, 1000 human genome dataset, NCBI dbSNP, and a database of 100 Chinese healthy adults. ExomeDepth software was used to detect copy number variation at the exon level.

dbNSFP (San Lucas et al., 2012), which contains seven well-established in silico prediction programs (MutationTaster, PhyloP, Scale-Invariant Feature Transform, LRT, and Polyphen2), was used to predict the effect of missense variants. Pathogenic variants were assessed according to the protocols issued by ACMG (Richards et al., 2015). The Human Gene Mutation Database (HGMD) was used to screen for mutations reported in published studies.

#### 2.2.3 Sanger Verification Method

All potential pathogenic variants and mutations were validated using conventional Sanger sequencing methods and compared to the normal human sequence in Genebank (NM_000102.3).

## 3 Results

After the variants were filtered, the correlation degree of the phenotype analysis was determined by “primary amenorrhea, hypokalemia, hypertension, and moyamoya disease” in which the values of the selected genes were significantly higher than those of other genes. Finally, four variants from *CYP17A1*, *CNOT3*, and *PCNT* were chosen. These variants were validated by Sanger sequencing and compared to the normal human sequence in GenBank (NM_000102.3, NM_014516.3, NM_006031.5). *CNOT3* and *PCNT* variants have not been reported in the HGMD database. The clinical diagnosis of MMD was confirmed based on the radiological evaluations (Scott and Smith, 2009). The diagnosis of 17OHD was supported by the *CYP17A1* mutation in conjunction with the findings of the biochemical and clinical evaluations.

### 3.1 *CYP17A1*

**FIGURE 1:**
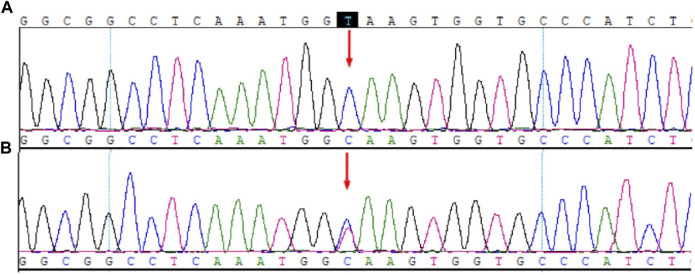
Mutation sites in *CYP17A1*. **(A)** Patient. **(B)** Father.

The results of WES and Sanger sequencing showed a homozygous intronic mutation c.297+2T>C (chr10-104596820 A>G, NM_000102.3, rs764723654) of *CYP17A1* (Figure 1). Variant c.297+2T>C is a classical splice site mutation containing a substitution of the T with C nucleotide at c.297+2. This mutation is pathogenic (PS3+PM2+PM3+PP4) according to the ACMG guidelines (Richards et al., 2015). This mutation was previously reported to cause reading frame shifts and premature termination, which eventually destroyed the activity of 17a-hydroxylase/17,20-lyase (Hwang et al., 2011). Sanger sequencing verified that the patient’s father was heterozygous for this mutation. The patient’s mother and brother could not provide blood samples; thus, their genetic information was missing.

### 3.2 *CNOT3*

**FIGURE 2:**
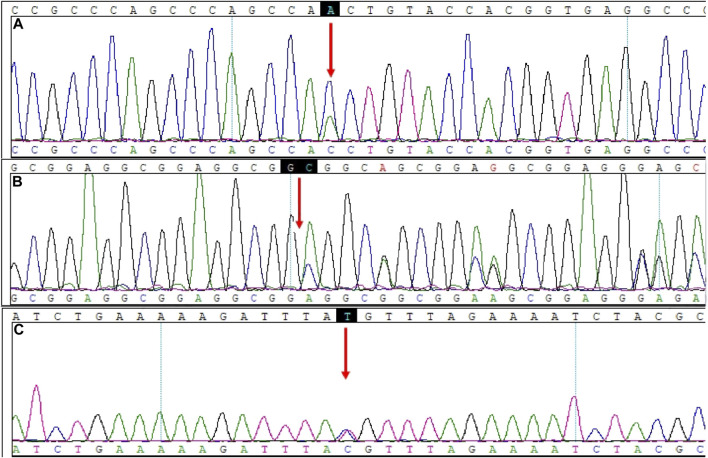
Mutation sites in *CNOT3* and *PCNT*. **(A)** *CNOT3*, NM_014516.3: c.827A>C. **(B)** *CNOT3*, NM_014516.3: c.1202_1203insAGGCGG. **(C)** *PCNT*, NM_006031.5: c.1069T>C.

The variants of CCR4-NOT transcription complex subunit 3 (*CNOT3*) found by WES and verified by Sanger sequencing included c.827A>C (chr19-54649769 A>C, NM_014516.3, p.Asn276Thr, rs201696237) and c.1202_1203insAGGCGG (chr19-54652174 A>AGCGGAG, NM_014516.3, p.Ser396_Gly397insGlyGly, rs771212010) (Figure 2).

The c.827A>C variant indicates a substitution of the A nucleotide with a C at c.827, causing a change from Asn276 to Thr276. The heterozygous variant c.827A>C was predicted to be neutral in the CONDEL Meta-prediction aggregator and suggested to be benign based on the Franklin ACMG Classification. PolyPhen2 software predicted that the p.Asn276Thr variant was benign, with a score of 0.001 (sensitivity: 99%; specificity: 9%). MutationTaster predictive analysis showed that the p.Asn276Thr variant may be a polymorphism, with a prediction accuracy of 65.7% (Table 2).

**TABLE 2:** *CNOT3* and *PCNT* variants.

Gene symbol	Transcript	cHGVS	pHGVS	Zygosity	Ens Condel Pred	ClinVar Significance	Franklin ACMG Classification	PolyPhen2	Mutation Taster	Filter	dbSNP Allele Freq	1000G AF	GnomAD AF	OMIM
*CNOT3*	NM_014516.3	c.827A>C	p.Asn276Thr	Het	Neutral	—	Likely benign	benign	polymorphism	PASS	<0.01	<0.01	<0.01	618672
		c.1202_1203insAGGCGG	p.Gly402_Ser403insGlyGly	Het	—	—	VUS	—	polymorphism	PASS	<0.01	—	<0.01	
*PCNT*	NM_006031.5	c.1069T>C	p.Cys357Arg	Het	Deleterious	Uncertain	VUS	benign	disease causing	PASS	<0.01	<0.01	<0.01	210720

cHGVS, variation of bases; pHGVS, variation of amino acid; Hom, homozygous; Het, heterozygous; Function, mutation type; Ens Condel Pred, results of CONDEL Meta-prediction aggregator; Filter, results of mutation quality control; ClinVar Significance, clinical significance of the mutation in ClinVar databases; Cds-ins, insertion in coding sequence; VUS, variation of uncertain significance; dbSNP Allele Freq, Allele frequency information of this SNP in dbSNP database; 1000G AF, Allele frequency information of this SNP in the 1000G_ALL database; GnomAD AF, Allele frequency information of this SNP in GnomAD database; OMIM, Phenotype OMIM number.

The c.1202_1203insAGGCGG variant indicates the insertion of nucleotides AGGCGG between nucleotide c.1202 and c.1203, causing a GlyGly insertion between Ser396 and Gly397. The heterozygous variant c.1202_1203insAGGCGG is suggested to be a variation of uncertain significance (VUS) based on the Franklin ACMG Classification. MutationTaster predictive analysis showed that the p.Ser396_Gly397insGlyGly variant may be a polymorphism, with a prediction accuracy of 99.9% (Table 2).

### 3.3 *PCNT*

The pericentrin (*PCNT*) variant found by WES and verified by Sanger sequencing was c.1069T > C (chr21-47768962 T>C, NM_006031.5, p.Cys357Arg, rs549300488) (Figure 2). This variant resulted in a change from Cys357 to Arg357. The CONDEL meta-prediction aggregator predicted that the c.1069T > C variant was deleterious, with uncertain clinical significance according to the ClinVar database. The Franklin ACMG Classification suggested the variant was VUS. The variant p.Cys357Arg was predicted to be benign, with a score of 0.111 (sensitivity: 91%; specificity: 69%) in PolyPhen2 but suggested to be disease-causing, with a prediction accuracy of 66.0% based on the MutationTaster predictive analysis (Table 2).

## 4 Discussion

The patient was diagnosed with 17OHD at 20 years of age based on the presence of amenorrhea, sexual infantilism, hypertension, and hypokalemia. She had a 46, XY karyotype and was homozygous for c.297+2T>C. This mutation was previously reported in China and Taiwan (Hwang et al., 2011; Dong et al., 2016; Li et al., 2020). However, none of these reported cases were associated with MMD. Hwang et al. (2011) detected the transcription of *CYP17A1* in patients by *in vitro* expression testing of an allogeneic minigene vector and reported that the mutation of this intron (c.297+2T>C) constituted a functional splicing site, resulting in the incorrect splicing of most of the mRNA transcripts (>90%) of *CYP17A1*. Through abnormal mRNA splicing into the pseudo-exons, the reading frame can be shifted and terminated prematurely, and the activity of 17a-hydroxylase/17,20-lyase is destroyed. Only correctly spliced products express normal exons and retain a fraction of the 17a-hydroxylase/17,20-lyase activity thus explaining the origin of the low but detectable serum cortisol levels, in this severely affected patient.

Deficient 17a-hydroxylase/17,20-lyase activity leads to the lack of formation of progesterone and 17a-hydroxylate pregnenolone, which prevents cortisol synthesis and accounts for elevated ACTH levels and overproduction of 17-deoxycorticosteroids by the zona fasciculata. Elevated corticosterone and deoxycorticosterone (DOC) levels lead to increased mineralocorticoid activity and subsequent sodium retention, volume expansion, and hypertension with suppressed renin and aldosterone (Auchus, 2001). At the same time, DOC causes increased potassium excretion from renal collecting tubules and subsequent hypokalemia (Zennaro et al., 2015). The patient was treated with oral dexamethasone (0.375 mg once daily), which led to an improvement in blood pressure, returned the potassium levels to the normal range, and suppressed corticosterone production.

However, the patient discontinued treatment 1 year later due to significant weight gain. Her blood pressure ranged from 150/110 to 160/120 mmHg after withdrawal. At 25 years of age, a left anterior communicating aneurysm and a bilateral middle cerebral artery occlusion were identified following sudden syncope. The patient was consequently diagnosed with MMD. MMD is a chronic and progressive occlusion cerebrovascular disease with unknown etiology. In Japan, about 10% of patients have affected first-degree relatives (Fukui et al., 2000). Associations with loci on chromosomes 17, 8, 6, and 3 in addition to specific HLA haplotypes, have previously been described (Fukui et al., 2000; Han et al., 2003; Sakurai et al., 2004).

In this patient, whole-exome sequencing revealed *CNOT3* and *PCNT* variants that might be related to MMD. Pinard et al. (2020) reported new mutations in *CNOT3* that were not previously associated with MMD and also identified them in unrelated MMD probands. *CNOT3* encodes proteins related to chromatin remodeling, together with *YY1AP1,* leading to MMD-like cerebrovascular occlusive disease, (Guo et al., 2017) suggesting that disrupted chromatin remodeling is a predisposing factor leading to early-onset non-atherosclerotic occlusive cerebrovascular disease. *PCNT* is located on chromosome 21, its diallelic dysfunction mutations can lead to microcephaly osteodysplasia primordial dwarfism type II disease (OMIM 210720), and about 50% of patients develop cerebrovascular abnormalities, including MMD, intracranial aneurysm (IAs), and subarachnoid hemorrhages (SAHs), as described by Bober et al. (2010). In addition, a previously described complete *Pcnt* knockout mouse model (*Pcnt* −/−) showed systemic vascular abnormalities, including intracranial hemorrhage (Chen et al., 2014), suggesting that *PCNT* is an important factor in MMD pathogenesis.

Although previous literature suggested that *CNOT3* and *PCNT* mutations may be pathogenic causes of MMD, the variants in the present patient were heterozygous with unknown significance; thus, further studies are needed. Moreover, *CNOT3*, *PCNT*, and *CYP17A1* are on different chromosomes, and *CYP17A1*, encoding the only cytochrome P450c17 enzyme, is only expressed in adrenal glands and gonads (Chung et al., 1987). Therefore, an association between 17OHD and MMD was not supported at the genetic level. However, Manolio et al. (2009) suggested that mutated heterozygous vectors in recessive genetic diseases may have led to an increased phenotypic risk of diseases or complex diseases. These findings require more research.

To characterize the relevance between MMD and 17OHD or Congenital Adrenal Hyperplasia (CAH), a literature review was performed. Three articles were identified from the PubMed, Web of Science, and CNKI China databases and are summarized in Table 3 (Wang et al., 2001; Gao et al., 2019; Vasudevan et al., 2019). As shown in Table 3, 17OHD with MMD is not an occasional case, and the locus of *CYP17A1* mutation is not associated with MMD. Thus, patients with 17OHD with no obvious neurological discomfort or severe hypertension would not receive cerebrovascular imaging examination. Consequently, they would not have the chance to be diagnosed with MMD.

**TABLE 3:** Previously reported cases of 17OHD or CAH with MMD.

Case	Diagnosis	Nucleotide changes	Karyotype	Age 1 (yr	Age 2 (yr)	Age 3 (yr)	BP (mmHg)	K (mmoL/L)	Neurological related PMH	Diagnostic procedure of MMD
1 (This case)	17OHD, MMD	c.297+2T>C (*CYP17A1*)	46,XY	20	25	28	160/120	3.0	(-)	Sudden loss of consciousness and blurred vision upon awakening. DSA found several cerebral artery occlusions, and moyamoya vessels were seen
2 (Wang et al., 2001)	17OHD, MMD	c.287G>T and c.1117delC (*CYP17A1*)	46,XX	16	16	16	150/95	3.2	Encephalitis at 10 years old	Middle cerebral artery occlusion was found by pituitary (MRI) during hospitalization after which MMD was further found by craniocerebral MRA.
3 (Vasudevan et al., 2019)	CAH, MMD	NA	46,XY	42	42	NA	NA	NA	(-)	Spontaneous ventricular hemorrhage and diagnosed as MMD. During hospitalization in neurosurgery department, found to has male sex development disorder, hypertension, and hypokalemia. Abdominal ultrasound suggested adrenal hyperplasia, lack of ovaries and uterus, and cryptorchidism in the groin
4 (Wang et al., 2001)	11β-OHD, MMD	NA	46,XY	5	9	6	175/110	1.9	Bilateral optic nerve atrophy	Cranial MRI suggested cerebral infarction during hospitalization, and MRA was further performed to find MMD.

Age 1, age at diagnosis of 17OHD; Age 2, age at diagnosis of MMD; Age 3, onset age of hypertension; BP, blood pressure; K, blood potassium; PMH, past medical history; 11β-OHD, 11β-hydroxylase deficiency; NA, not available; DSA, digital subtraction angiography; MRI, magnetic resonance imaging; MMD, moyamoya disease; MRA, magnetic resonance angiography.

Several genetic syndromes, such as neurofibromatosis type I (OMIM 162200) have been described by Friedman et al. (2002), while Moyamoya angiopathy with achalasia and hypertension (OMIM 615750) was described by Herve et al. (2014). These genetic findings highlight potential molecular pathways, including the deletion of nitric oxide signaling (*GUCY1A3*), that could account for MMD (Herve et al., 2014) and activation of proliferative pathways (*NF1*) (Friedman et al., 2002). Therefore, 17OHD may also be a risk factor for MMD. One hypothesis proposed that hypertension and hypokalemia caused by increased deoxycorticosterone levels due to *CYP17A1* defects may play a dominant role in the pathogenesis of MMD, which requires further exploration. Several previous cases also mentioned that patients with 17OHD had a history of cerebral hemorrhage or cerebral ischemia (Mita et al., 1983; Yu et al., 2010; Zhang et al., 2021). No clear correlation in genetic levels between 17OHD and MMD in patients with 17OHD and hypertension was found. However, the risk of cerebrovascular accidents should be considered.

The coexistence of 17OHD and MMD in one person is extremely rare. No other study on the genetic analysis of 17OHD accompanied by MMD has been published. Cerebral ischemia and hemorrhage are important risks affecting the quality of life and prognosis of patients with 17OHD. Therefore, cerebrovascular examination in these patients may help to prevent life-threatening intracranial vascular disease. Early diagnosis allows the safe administration of treatments such as revascularization and can prevent or reduce long-term sequelae in these patients.

## Data Availability

The datasets for this article are not publicly available due to concerns regarding participant/patient anonymity. Requests to access the datasets should be directed to the corresponding author.
